# Management practices and welfare of working equids in Mogalakwena local municipality, Limpopo Province, South Africa

**DOI:** 10.1017/awf.2026.10097

**Published:** 2026-07-23

**Authors:** Dimakatso Betty Molapo, Cheryl M.E. McCrindle, Tulisiwe Pilisiwe Mbombo-Dweba, James Wabwire Oguttu

**Affiliations:** Department of Agriculture and Animal Health, University of South Africa College of Agriculture and Environmental Sciences, South Africa

**Keywords:** Animal welfare, donkeys, equine owners, livelihoods, social position, supplementary feeding, veterinary services

## Abstract

This paper describes the management and welfare aspects of Working Equids (WEs) in Limpopo Province, South Africa by utilising a cross-sectional study design with data collected via a semi-structured questionnaire. All households with WEs were invited to participate in the study, and 111 volunteered to take part. Approximately half (50; 45%) of respondents did not have a regular feeding schedule for their WEs, while 92 (79%) did not provide supplementary feeding during times when pastures were poor. None of the equid owners (EOs) surveyed had their equids vaccinated while 88.29 and 53.15% failed to provide treatment for internal and external parasites, respectively. Most respondents (79.28%) treated sick animals themselves with 6.31% doing nothing when the animal was ill. A small proportion (5.41%) expressed the sentiment that equids, in particular donkeys, do not get sick. Most (77.48%), on a daily basis worked their equids for 3–6 h, while 2.7% worked them for 7–11 h. Over half (57.66%) indicated that per day their equids covered 0–4 km per day, with only 2.7% reporting that their equids travelled more than 10 km per day. Although 99.10% of respondents allowed their equids to rest during the day, loads remained on the animals during rest time. Within our study area WEs are exposed to the negative effects of inadequate management, and negative stereotypes towards equids, especially donkeys, persist among EOs. The welfare of WEs needs to be prioritised in order for their well-being to improve.

## Introduction

Previously, Working Equids (WEs) were prevalent throughout the world, with them employed in a variety of different tasks. Technological advancements helped determine the extent of their withdrawal from functioning as modes of transportation (Haddy *et al.* 2020; Mota-Rojas *et al.* 2021; Norris *et al.* 2021). However, despite animal traction and draught being phased out of South Africa’s commercial industries, previous research carried out in the country’s nine provinces has shown smallholder farmers and rural communities continue to use it extensively (O’Neill *et al.* 1999; Letsoalo *et al.* 2000; Wels & Krecek 2001). In fact, it is estimated that worldwide there are millions of people who still depend upon WEs for their livelihoods (Sommerville *et al.* 2018; Cameron *et al.* 2026).

The use of equids within rural and peri-urban communities is linked to poverty alleviation, sustaining livelihoods, and building social capital among equid-owning households and their communities. Social capital refers to the networks, relationships, and norms that facilitate cooperation and collective action for mutual benefit (Woolcock & Narayan 2000). Through social networks characterised by trust, reciprocity, and cooperation, households can gain access to resources, information, and social support that strengthen resilience and enhance their capacity to cope with economic challenges (Szreter & Woolcock 2004). In this context, social capital strengthens community support systems, enhancing the ability of households to cope with economic challenges and improve their well-being.

WEs, where they are utilised, contribute substantially to socioeconomic well-being through helping communities with domestic transportation requirements, by creating employment and acting as a source of income (Grace *et al.* 2022). WEs also contribute via reducing human labour. Moreover, they play a vital role in helping attain various UN Sustainable Development Goals (SDGs): fetching water and food production (SDG 1); providing edible products (SDG 2); accessing health services and products (SDG 3 and 5) and benefitting women (SDG 6) (Grace *et al.* 2022).

The term ‘animal welfare’ describes the mental and physical condition of animals that are influenced by their living and working environments, by human beliefs and behaviours and by the resources available to them (Arega *et al.* 2023). Broom (2016) described animal welfare as the welfare of an individual animal with regards to its attempts to cope with its environment.

Since the discussion of animal welfare started in the 1960s, methods for conceptualising and assessing animal welfare have undergone significant change. The field of animal welfare has evolved from the foundational framework of the Five Freedoms to the more comprehensive Five Domains model. The Five Freedoms outline the basic requirements for animals to express natural behaviours and avoid stressful conditions that negatively impact welfare (Webster 2016). While these freedoms establish essential baseline standards, the Five Domains model advances this perspective by offering a holistic approach that not only focuses on mitigating negative states but also emphasises the promotion of positive physical, behavioural, and mental experiences in animals (Mellor *et al.* 2020). This evolution reflects a broader understanding of welfare that integrates both the alleviation of suffering and the enhancement of animals’ quality of life. The welfare of WEs is particularly important, not only for the health and survival of the animals themselves but also for the livelihoods of the people who depend on them (Tesfaye & Martin Curran 2005; Grace *et al.* 2022).

Relevant laws are in place in many nations that are aimed at safeguarding the welfare of WEs (FAO 2014), but there are few efficient enforcement mechanisms. Similarly, the South African government has the Animal Protection Act (APA), Act 71 of 1962. This is a law that governs the protection of animals in South Africa, including working animals in relation to the majority of issues related to the prevention of animal cruelty (Animal Protection Act 1962). Furthermore, the National Society for the Prevention of Cruelty to Animals (NSPCA) was established in 1955, to provide a forum to the uniformity of welfare legislation and standards (Societies for the Prevention of Cruelty to Animals Act 1993). Additionally, the NSPCA was established as a statutory body to deal with national issues and enforce the Animal Protection Act No. 71 of 1962 throughout South Africa (Societies for the Prevention of Cruelty to Animals Act 1993). The Animal Protection Act 71 of 1962, stipulates that if an animal owner violates any provisions of this act, they are liable to be prosecuted under the relevant sections applicable to the case (Animal Protection Act 1962).

However, WEs globally continue to experience compromised welfare due to inadequate management practices, low social status, insufficient nutrition, and ill health (Fasil & Yenewhunegnaw 2017; Njisane *et al.* 2020; Derbib *et al.* 2024). Despite the vital contribution they make to people’s livelihoods, husbandry practices often remain inadequate (Haddy *et al.* 2020). Working equids frequently operate under harsh conditions that limit their productivity and see them face similar welfare challenges irrespective of whether they are used for commercial or domestic purposes (Valette 2015). International reports, including those by the FAO (2014), have highlighted the persistent lack of attention paid to WE welfare at international, national, and local levels, as well as the limited capacity of owners to meet basic welfare needs. More recent studies have continued to document compromised welfare linked to poor living conditions, limited access to veterinary services, and broader socioeconomic constraints (Bonsi *et al.* 2023; Derbib *et al.* 2024; Merridale-Punter *et al.* 2024). Available evidence further suggests that education and community-based interventions can improve welfare outcomes, underscoring the need for sustained policy engagement and targeted support for working equid-owning communities (Sommerville *et al.* 2018; Grace *et al.* 2022).

Moreover, there is evidence that also suggests the welfare of WEs receives little to no attention from policy-makers (Bonsi *et al.* 2023) and professional bodies (Langkos 2014). A systematic review of studies conducted between 2010–2024 concluded that research on welfare of WEs is also lacking (Tariku *et al.* 2025). In support of this, Merridale-Punter *et al.* (2024) added that published studies on the epidemiology and health and welfare of WEs is limited. There is no evidence of research that clarifies the magnitude of these issues in South Africa, particularly in the Mogalakwena Local Municipality, Limpopo Province, South Africa. Previous studies have argued that addressing welfare issues of WEs requires tailored interventions that are sensitive to local geo-cultural and socio-economic attitudes (Luna *et al.* 2017; Haddy *et al.* 2022).

Our study sought to address the fact that very few recent publications exist regarding the management practices and welfare aspects of WEs in the rural and peri-urban areas in Mogalakwena Local Municipality, Limpopo Province of South Africa, or even in the whole of South Africa. Such information is crucial to help cultivate better strategies for improving the health and welfare of WEs. Furthermore, information on management and welfare status can assist in finding methods to improve the utilisation and management of WEs.

## Materials and methods

### Ethical considerations

Ethical approval was obtained from the Research Ethics Committee of the University of South Africa (2021/CAES_HREC/141) prior to the onset of the study. Informed consent was provided for data usage from participants as per the ethical guidelines of the University of South Africa.

### Study area

**Figure 1.**
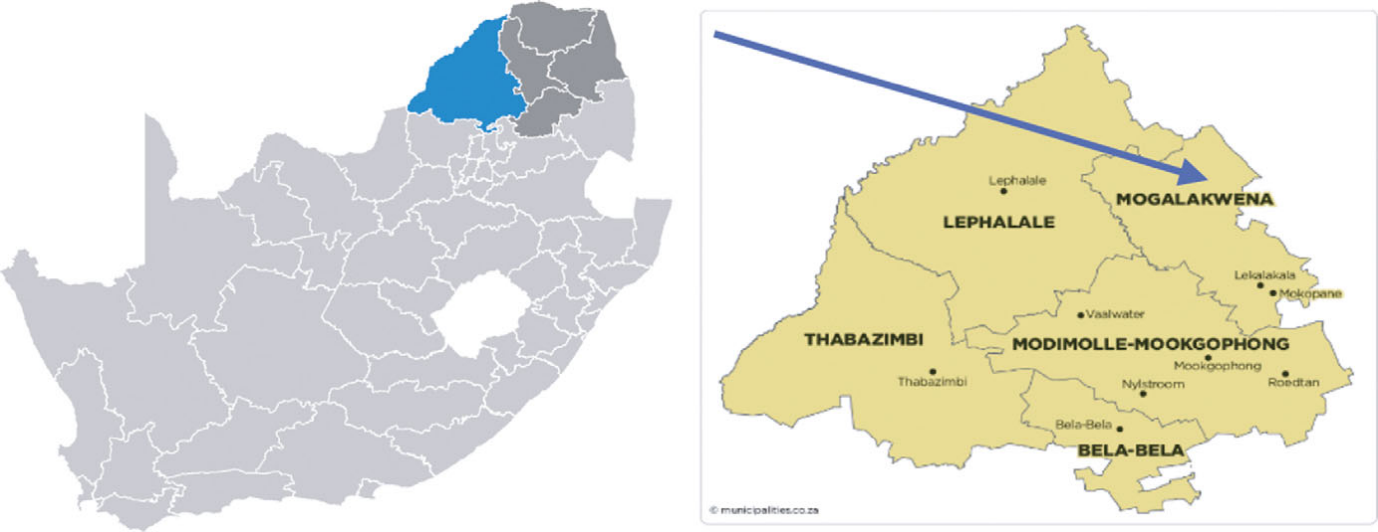
Geographic location of the study area within the Waterberg District Municipality, Limpopo Province, South Africa, showing Mogalakwena local municipality where household surveys of working equid owners (n = 111 households) were conducted. Figure 1. long description.

The Mogalakwena Local Municipality, located in the Waterberg District, Limpopo Province (Figure 1), accounts for 45% of the total population of Waterberg District (Mogalakwena Local Municipalty 2020). The Mogalakwena Local Municipality, like the majority of rural municipalities in the Republic of South Africa, is characterised by unemployment and poverty (Mogalakwena Local Municipality 2019). In Mogalakwena Local Municipality, unemployment ranges from 45 to 70%, making poverty in the area, a serious social issue (Mogalakwena Local Municipality 2020). The local economy is based on mining and agriculture. Although the Municipality offers a variety of transportation options, donkey carts are among the most popular means of transport in the area (Mogalakwena Local Municipality 2018). The municipality experiences hot, pleasant summers and mild winters with temperatures ranging from 27 to the mid 34°C (Mogalakwena Local Mucipality 2019). The topography of the area is characterised by irregular undulating lowlands with hills and low-lying mountains.

### Study population

The study population consisted of all identified households in the 14 peri-urban areas of Mogalakwena local municipality, that owned WEs. Heads of household, irrespective of age or gender; or alternative adults (> 18 years) were invited to participate. One consenting member per household was interviewed, with participation voluntary and free of charge.

### Household selection

A ‘snowball’ sampling method as described by Valerio *et al.* (2016) was adopted to identify households within each selected peri-urban area that owned WEs. The snowball sampling method was adopted as no official list of households owning WEs within the selected study area existed and the units under study were not easily identifiable. As a result, interviewers established contact with a couple of households that owned any species of WEs before requesting that helped identify the next household with WEs. The new households would then be asked to nominate other households until all known households had been identified.

### Study design

This study used both qualitative and quantitative research methodologies. Data were also gathered using a one-on-one semi-structured face-to-face interview, via a pre-tested questionnaire that sought to collect information on owners’ demographics, management practices and welfare of WEs (see SI; Supplementary Material).

### Data collection

Internal validity of the data collection instrument was established through expert review prior to data collection. Face-to-face structured interviews were conducted with the owners of WEs by the principal researcher and research assistants (Animal health students with a higher certificate in Animal Welfare). Interviews lasted between 30–45 min. The research assistants were trained in interviewing techniques, questionnaire administration and data recording by the principal researcher. Data were collected manually.

### Data management

Questionnaires were assessed for completeness prior to data capture with all having been completed. Data were then assessed for inconsistences and missing information before being cleaned and coded using Microsoft Excel® Version 2016.

### Data analysis

Data were analysed using Microsoft Excel® Version 2016 and STATA (Version 17) with descriptive statistics computed as percentages or proportions, in the case of categorical variables. Continuous variables were assessed for normality using graphical methods, including histograms and normal probability (Q–Q) plots (Wilk & Gnanadesikan 1968). This assessment informed the choice of appropriate summary measures (means or medians).

## Results and Discussion

### Demographics

The study focused on 14 peri-urban areas within the Mogalakwena local municipality, which has a total population of approximately 307,682 people living in 79,396 households, representing 45% of the Waterberg District population (Census 2011). It should be noted that both the total population and household figure cover the entire municipality, including urban, rural, and peri-urban areas.

The number of participants who owned traction animals and agreed to voluntarily participate in the study from each area is presented in SII in the Supplementary Material. A total of 111 households that owned WEs participated in the study. The highest proportion of owners originated from Sekgakgapeng (n = 19; 17.1%); followed by Maroteng (n = 17; 15.3%), Tshamahansi (n = 12; 10.8%) and Mosesetjane (n = 13; 11.7%). Two areas (Mitchel and Mountain View) did not have households that owned WEs.

During the study, 407 WEs were recorded, with 99.3% donkeys and 0.7% mules; no horses were found (see SIII in the Supplementary Material). Donkeys were deemed preferable due to affordability, ease of maintenance, and perceived resistance to illness. Mules, although recognised for strength, longevity, intelligence, endurance, good hooves, and lower feed needs, tend to be viewed as aggressive and hard to train. Moreover, most respondents noted a reduced availability of mules and horses for purchase as well as issues with high prices.

### Management of working equines

#### Feeding management

The frequency of grazing WEs varied considerably among respondents (Table 1). Thirty-seven percent of owners (n = 41; 36.9%) reported allowing their equids to graze once daily at a specific time. Additionally, 8.1% (n = 9) indicated that grazing occurred twice daily, typically in multiple short sessions, such as in the morning and again after work in the afternoon. In contrast, 50.5% (n = 56) did not adhere to a regular grazing schedule; instead, equids grazed immediately after work, overnight, or intermittently throughout the day when not working.

**Table 1.** Feeding practices, water sources, and night-time housing management of working equids (predominantly donkeys [*Equus asinus*]) owned by households (n = 111) in peri-urban areas of Mogalakwena Local Municipality, Limpopo Province, South Africa. Percentages may exceed 100% where respondents selected multiple responses. Table 1. long description.

Variable	Level	Frequency(n)	Percentages(%)
How often do you take your equids out for grazing?	Once	41	36.94
	Twice	9	8.11
	Three times	5	4.50
	No grazing schedule	56	50.45
Do you provide supplementary feeding to your equines?	No	103	92.79
	Yes	8	7.21
What do your equids feed mainly on?	Crop residues	19	17.12
	Grazing	106	95.50
	Concentrates	3	2.70
	Other	2	1.80
From where your equids get drinking water?	Kraal	97	87.39
	Stream	18	16.22
Where do you keep your equids at night?	Kraal	50	45.05
	Bush	62	55.86
	Around the yard	7	6.31

While the reported feeding practices suggest the potential for nutritional deficiency, this study did not assess feed quantities, nutritional composition, grazing duration, or workload. Nevertheless, restricted grazing time due to work schedules and confinement has been widely reported and may increase the risk of compromised welfare and inadequate nutritional intake (Burn *et al.* 2010; Bukhari *et al.* 2023; Bihon *et al.* 2025).

As shown in Table 1, the majority of respondents (95.4%; n = 106) reported relying on grazing as the primary source of nutrition for their equids. In addition, some equids were fed crop residues and concentrates, while 1.80% of respondents (n = 2) indicated that they provided kitchen residues and leftovers. Although these feeding practices may indicate potential limitations nutritionally, diet adequacy cannot undergo direct evaluation within the scope of this study. Nonetheless, access to adequate quantities of good-quality feed is widely recognised as important for maintaining optimal body condition and meeting the increased energy demands associated with work, reproduction, and offspring rearing (Molla *et al.* 2017; Masebo *et al.* 2025).

In the present study, a large majority of respondents (n = 103; 92.79%) reported not providing feed supplements, relying solely instead on free grazing, even during winter months when pasture quality is typically reduced (Table 1). Similar patterns have been reported elsewhere, for example, Biffa and Woldemeskel (2006) found that horses and donkeys in Ethiopia were generally not provided with supplemental feed. Likewise, Swai and Bwanga (2008) reported that despite more than 95% of respondents in Tanzania allowing donkeys to graze freely, only 15% supplemented their equids with protein- and energy-rich diets. In South Africa, Marufu (2014) similarly observed that while most working equid owners in the Eastern Cape relied on free grazing, only 17% provided feed enhancement.

Although this study did not directly assess nutritional status, previous research suggests that the absence of supplementation during periods of forage scarcity may be associated with increased welfare risks. Prolonged reliance on poor-quality pasture has been linked to compromised body condition, reduced work capacity, impaired immune function, and increased susceptibility to disease (Burn *et al.* 2010).

A critical aspect of the health and performance of working equids is water intake and as shown in Table 1, the majority of EOs (n = 97; 87.4%) reported providing water to their WEs in buckets placed inside enclosures either during the daytime or night, typically before or after work. While 16.2% (n = 18) reported that equids left out in the bushes or simply to graze after working had to fend for themselves and drink from streams and/or rivers. It is important to note that the quantity and quality of water consumed can significantly influence the health and working capacity of equids. Dehydration due to inadequate water intake can lead to decreased physical performance, impaired thermoregulation, and increased susceptibility to heat stress and other health complications (Burn *et al.* 2010; Urošević *et al.* 2025).

### Housing management

Just under half of participants (n = 50; 45.05%) reported housing their working equids in a kraal, also referred to as ‘lesaka’ or ‘isibaya’ in certain African languages. A kraal is a traditional livestock enclosure typically constructed within African homesteads using locally available materials, such as wood or thorn bushes (Banda & Van Der Merwe 2017; Russell 2022). These enclosures are designed to protect livestock from predators and theft during night-time but offer minimal shelter from environmental elements. James and Krecek (1999) reported the necessity, in South Africa, of keeping livestock in kraals to guard against theft and predation. Similarly, Amante *et al.* (2014) reported that over three-quarters of respondents (76.6%) provided shelter for their WEs during the night to protect them from predators or other factors. The remaining 55.9% (n = 62) of EOs in this study, indicated that they allowed their equines to roam freely at night. Only a few (n = 7; 6.3%) reported keeping equids at home in their yards during the night.

Dressie *et al.* (2017), in Bahir Dar Town, Northwest Ethiopia, reported that 86% (n = 136) of respondents constructed houses for their mules to provide protection from rainfall and sunlight. This contrasts with the findings of our study which tended to indicate that WEs were primarily kept in kraals or around owners’ yards, offering minimal to zero protection from environmental elements. Besides suggesting that welfare of WEs in South Africa falls short of the standards seen in other parts of sub-Saharan Africa, such practices are in direct contravention of animal welfare laws, such as the Animal Protection Act (1962). The latter stipulates that animals must be afforded shelter to ensure safety from a variety of risks, including inclement and extreme weather, insects, theft, wild animals, and road traffic accidents.

### Disease management and prevention

#### Veterinary care

It is not uncommon to find EOs providing healthcare for their WEs. For example, in the study area, a large majority (79.3%; n = 88) of respondents treated their WEs themselves as opposed to calling a veterinarian (Table 2). This high prevalence has important implications as regards the health and welfare of the animals in question. Without appropriate diagnosis and treatment by trained veterinarians, such WEs are at an increased risk of suffering from untreated or improperly managed diseases (Pritchard *et al.* 2005). Moreover, inaccurate diagnosis can lead to the misuse or overuse of medications, increasing the risk of antimicrobial resistance and adverse side-effects (Burn *et al.* 2010; Nye *et al.* 2020).

**Table 2.** Disease management, access to veterinary medicines, and parasite control practices for working equids (mainly donkeys [*Equus asinus*]) as reported by equid-owning households (n = 111) in Mogalakwena Local Municipality, Limpopo Province, South Africa. Table 2. long description.

Variable	Action	Frequency (n)	Percentages (%)
What do you do when your equid is sick?	Call local welfare organisation / vet	8	7.21
	Treat it myself	88	79.28
	Call another equids owner	2	1.80
	Do nothing	7	6.31
	They don’t get sick	6	5.41
If you need drugs, where do you get them?			
Agricultural co-operative (co-op)	Yes	75	67.57
Local Society for the Prevention of Cruelty to Animals	Yes	4	3.60
Get them from the neighbour	Yes	5	4.50
I do not buy medicine for equids	Yes	32	28.83
What types of drugs do you commonly buy?			
Antiparasitic drugs	Yes	55	49.55
Antibiotics	Yes	1	0.90
Antiseptics	Yes	41	36.94
Not sure	Yes	13	11.71
Do you treat your equids for internal parasites?	No	98	88.29
	Yes	13	11.71
Do you treat equids for external parasites?	No	59	53.15
	Yes	52	46.85
Do you vaccinate your equids for diseases?	No	111	100
	Yes	0	0
Which of the following have you performed on your equids in the last three months?	Castration	1	0.90
	Hoof trimming	0	0
	Dentistry	0	0

With respect to the attitude towards sick animals, as shown in Table 2, 6.3% (n = 7) of respondents indicated that they would do nothing if their WEs were sick, while 1.8% (n = 2) said that they would seek help from other equid owners. However, 5.4% (n = 6) were of the opinion that equids, especially donkeys, did not get sick, an attitude that has been observed elsewhere. For example, in studies conducted in South Africa, Biffa and Woldemeskel (2006) reported that 39.3% of respondents did not treat their horses or donkeys, irrespective of the prevalence and severity of injuries. The belief that equids are beasts of burden and do not become ill is a major contributing factor to the limited provision of veterinary treatment in equid-owning communities, alongside other socioeconomic constraints, such as limited financial resources, poor access to veterinary services, and inadequate knowledge of equid health and welfare needs.

Among the 111 respondents, 88.3% (n = 98) and 53.2% (n = 59) reported not treating their equids for internal and external parasites, respectively, while 11.7% (n = 13) and 46.8% (n = 52) reported treating their equids. According to the Animal Diseases Act (1984) and the Animal Protection Act (1962), animal owners are responsible for ensuring their animals receive appropriate treatment for parasitic infestations. Notably, among those who treated their WEs for external or internal parasites, most did so without supervision from a veterinary official or authorised personnel. This raises concerns, as the Animal Diseases Act (1984), Section 11, stipulates that only authorised personnel, such as state veterinarians and animal health technicians, are permitted to administer medication. Unsupervised treatment can lead to over- or underdosing, improper drug use, and subsequent development of parasite resistance (Sangster 2001), which poses a significant threat both to animal health and that of the wider veterinary community.

A large percentage of respondents (67.6%; n = 75) identified a nearby agriculture co-operative (Co-op), as being the likely source of veterinary medicine when required. This is a positive finding, as it suggests that equid owners (EOs) know where to access reliable sources of veterinary medicines for their animals. However, 4.5% (n = 5) indicated their neighbours as the likeliest port of call should they need medication for their equids; especially those owning livestock (n = 4; 3.6%) or they would get medicines from local welfare organisations. The latter two practices could lead to under- or over-exposure to medication, which could predispose the animals to risks associated with either under- or overdosing. Half (n = 55; 49.6%) of the respondents reported purchasing antiparasitic drugs and only (n = 41; 36.9%) reported that they bought antiseptics. However, thirteen (11.7%) were unsure of the kind of medication they commonly bought (Table 2). Those not buying medication, indicated that they applied used car oil and battery ash to wounds as well as ‘Vaseline’ (Vaseline ® White Petroleum Jelly) and ‘Jeyes Fluid’® (Jeyes Group Ltd) – a quaternary ammonium compound used for disinfection and for tick control. Similar patterns were observed by Nye *et al.* (2021) in Northern India where 62% (n = 23) of owners relied first upon themselves to treat their animals with home remedies or traditional medicine. These alternatives could increase the risk of wound infections or raise the possibility of hazardous exposure.

It was also observed that none of the respondents, provided dental or hoof care to their animals. Further probing revealed that this was due to lack of both knowledge and service providers. Only one (0.9%) respondent indicated that he performed castration on his equids, while the rest (n = 110; 99.1%) did not. The reason not to castrate equids was given as male equids being used for breeding. The one individual who did report castrating his male equids, did so mainly to prevent fighting amongst stallions. Similar findings were reported by Taylor (1999) in the former Ciskei region of the Eastern Cape, South Africa, where equid owners castrated their animals to restrict breeding and address behavioural issues. However, the negative aspect to this practice of self-castration is that sedatives are very seldom used, resulting in severe pain.

While disease prevention is a key component of animal welfare, findings from the present study indicate that the WEs’ health was deemed a low priority in the study area. This is supported by the fact that no respondents reported vaccinating their equids against any disease. This could be attributed to the widely held view that equids, especially donkeys, are extremely resilient and resistant to diseases (Starkey 1995). Disease-prevention strategies, including vaccination programmes, are essential for WEs but were notably absent in the study area.

According to all respondents (n = 111; 100%) who participated in the study, there were no governmental interventions or services related to equids in the vicinity (see SIV in the Supplementary Material). However, 16.2% (n = 18) reported that there were organisations/institutions that were concerned with equid welfare in their area. When asked whether they had received any assistance from said organisations, 9.9% (n = 11) indicated that in the three months leading up to the interview they had received nothing.

Only 3.6% (n = 4) of the participants reported having received veterinary services while 0.9% (n = 1) indicated that impounding of stray or free-roaming animals was offered as part of the veterinary services. In fact impoundment of animals is provided for by law in the country (Bergrivier Municipality 2009). Others indicated euthanasia as being one of the services offered as part of the veterinary services in the study area. According to de Klerk *et al.* (2020), in the Cape Flats, South Africa, there are animal welfare organisations, such as the Cart Horse Protection Association (CHPA) that oversee the welfare of WEs. A study by Marufu (2014) based in the peri-urban areas of the Eastern Cape, South Africa, reported veterinary treatment and training for donkey care only being provided by two animal welfare organisations in the region. However, the results of the current study diverge notably from these reports, as a significant majority (n = 93; 83.8%) stated that no organisations or institutions dedicated to equid welfare existed in their areas. This suggests a substantial gap in access to animal welfare organisations and governmental institutions offering veterinary care and training for WEs within the communities surveyed. It is also possible that owners are simply unaware of such services, contributing to the perceived lack of access. Most of the respondents in the peri-urban areas of Mogalakwena attributed the lack of veterinary care to financial challenges and a lack of resources in their communities, including veterinary clinics and veterinary services. This echoes observations elsewhere in the country. For example, James and Krecek (1999) in their South African study also reported a lack of access to veterinarians, especially in rural regions, coupled with financial difficulties, resulting in many people not being able to access to veterinary care.

### Working schedules of working equines

WEs were used in a variety of sectors in the study region to generate income for the owners. As a result, the frequency, nature, and timing of the use of WEs varied depending on the season (Table 3). These findings corroborated reports suggesting that season has an effect on the variety of work done by WEs (de Klerk *et al.* 2020). Seasonal changes were observed regarding the frequency of WE use in the study area, with winter being the peak time when WEs were utilised mostly for carting. This might be explained by increased demand for firewood during the cold season and the current loadshedding experienced in South Africa. A similar observation was made by Marufu (2014) who noted that a high percentage (84.51%) of WE owners in the Eastern Cape described winter as the donkey’s busiest time of year for carrying firewood. However, these findings contrast with those of Wells and Krecek (2001), who reported that working equids in Moretele 1, South Africa, expended greater effort during the ploughing season, which runs from July to September. In a study conducted in Cape Town, de Klerk *et al.* (2020) observed the significant impact of season on the ability of WEs to carry out their work. Half of the respondents in their study reported working less in the winter with 32 citing seasonal rains as a contributing factor.

**Table 3.** Seasonal patterns of use, daily working hours, distance travelled, rest breaks, and communication methods used with working equids (mainly donkeys [*Equus asinus*]) as reported by equid-owning households (n = 111) in Mogalakwena Local Municipality, Limpopo Province, South Africa Table 3. long description.

Variable	Level	Frequency (n)	Percentage (%)
How often do you use your equines?	Seasonal	3	2.70
	Daily	33	29.73
	Weekly	6	5.41
	Availability of work	68	61.26
	Not Applicable	1	0.90
In which season are your equines mostly used?			
Summer	No	42	37.84
	Yes	68	61.26
	Not applicable	1	0.90
Winter	No	31	27.93
	Yes	79	71.17
	Not applicable	1	0.90
Spring	No	81	72.97
	Yes	29	26.13
	Not applicable	1	0.90
Autumn	No	85	76.58
	Yes	25	22.52
	Not applicable	1	0.90
On average, how many hours do you work equines in a day?	Less than 2 h	21	18.92
	3 – 6 h	86	77.48
	7–11 h	3	2.70
	More than 11 h	0	0
	Depends on number of loads	1	0.90
How long do your equines travel per day?	0 – 4 km	64	57.66
	5 – 10 km	44	39.64
	More than 10 kms	3	2.70
When working with your equines do you give them breaks?	No	0	0
	Yes	110	99.10
	Not Applicable	1	0.90
How do you communicate with equines when working?			
Voice command	No	6	5.41
	Yes	105	94.59
Whipping	No	99	89.19
	Yes	12	10.81
Pull in the reins	No	99	89.19
	Yes	12	10.81

The work schedule for WEs varied extensively in the study area. For example, 61.3% (n = 68) of respondents described their work schedule as being based on the availability of clients or work. They indicated that certain times of the month, such as pension days and/or month end is when there is a greater need for transportation from various peri-urban areas to the city, where everyone buys their necessities. Approximately 30% (n = 28; 29.7%) of equid owners in the study area indicated making use of their equids on a daily basis while a small percentage (n = 3; 2.7%) only did so on a seasonal basis.

Over three-quarters (77.5%; n = 86) of respondents worked their equines for 3–6 h per day, while 18.9% (n = 21) did so for less than 2 h per day (Table 3). Overworking and inadequate resting periods of WEs may be a common problem in the current study because the majority of respondents indicated an increase in working hours in response to the needs of the clients and/or to increase income. This was evident from the 2.7% (n = 3) of WE owners in the study area who reported working their equines between 7–11 h each day while 39.6% (n = 44) indicated that their WEs travel between 5–10 km per day when at work. These findings contravene the ‘prevention of cruelty to draught and pack animals’ rules” (FAO 1968), which state no person shall use any animal for drawing any vehicle or carrying any load for more than an average of 9 h per day.

Almost all the respondents (n = 110; 99.1%) described providing their equines with a rest break during the working day with the load left on the animal (Table 3). Although the majority of WE owners indicated that they gave their equines rests in-between duties, some noted that on days when business was booming, this was not always the case. This has potential to result in fatigued animals. A number of respondents revealed that equids were not provided water, food, or shade during rest breaks. Other authors have also reported poor care and management of WEs, for example, Haddy *et al.* (2021) observed only 6% of WEs had free access to a shade, and 59% had no access to water.

A variety of methods of communication were described by owners as regards their equids during the driving of equine carts, including voice commands, whipping and tugging the reins. Among respondents interviewed, 10.8% (n = 12) reported whipping their equids (Table 3). These actions are in direct contravention of the Animal Protection Act 71 of 1962 section 2(1)a, which prohibits such acts as overloading, overriding, overdriving, beating, kicking, goading, ill-treatment, neglect, infuriating, terrifying, torturing, or maiming of any animal.

Responsible equid management and welfare frequently focus upon the details of care that go into providing animals with a healthy life, without forgetting that owners of WEs are under obligation to ensure that animals should not be denied their fundamental Five Freedoms when they come to the end of their working life. In view of this, it was worth noting that 79.3% (n = 88) of our respondents were in favour of keeping their WEs, ensuring they are cared for humanely, even beyond their working lives (Table 4). Only 6.3% (n = 7) of respondents indicated that they were willing to surrender their equids to their local SPCA, while only one respondent reported that they would euthanise their working equids. Furthermore, 4.5% (n = 5) of respondents indicated that they frequently provided insufficient care to their working equids once they were no longer able to work. Such practices are discouraged by WOAH (2019), which recommends that consideration be given at the end of an equid’s working life “*to avoid the equids suffering a prolonged and painful death by abandonment, neglect or disease, painful death such as being eaten by wild animals, or hit by a motor vehicle*”.

**Table 4.** Reported fate of working equids (mainly donkeys, [*Equus asinus*]) at the end of their working lives, based on responses from equid-owning households (n = 111) in Mogalakwena Local Municipality, Limpopo Province, South Africa Table 4. long description.

Variable	Option	Frequency (n)	Percentages %
What happens to your equids if it can’t work anymore?	Euthanasia	1	0.90
	Sell it	4	3.60
	Continue taking care until it dies	88	79.28
	Slaughter for meat	0	0
	Take it to Local SPCA	7	6.31
	Neglect	5	4.50

### Harnessing

Properly designed and fitted harnesses allow WEs to pull carts to the best of their ability, with limited risk of being injured. This study revealed that majority of owners (n = 110; 99.1%) harness their equids during working, with 51.4% (n = 57) reporting the harnesses in question as being inappropriate for work (Table 5). This self-reported figure may be an underestimation of the true prevalence of harness-related issues since the remaining harnesses may also be suboptimal. In addition, the harnesses in use tended to be constructed from inappropriate materials, such as discarded safety vehicle belts, old clothing, ropes, and conveyor belts.

**Table 5.** Use and perceived suitability of harnessing equipment for working equids (mainly donkeys [*Equus asinus*]) as reported by equid owners (n = 111 households) in Mogalakwena Local Municipality, Limpopo Province, South Africa Table 5. long description.

Variable	Level	Frequency (n)	Percentages %
Do you use harness for work?	No	1	0.90
	Yes	110	99.10
Is it appropriate for your type of work?	No	53	47.75
	Yes	57	51.35

These findings are consistent with those of Hanekom (1995) who conducted a study in South Africa reporting frequent use of ropes and conveyor belts by farmers with wires often used for fixation, leading to bruising and chafing. Biffa and Woldemeskel (2006) also observed similar findings whereby car tyre strips were utilised as equid harnesses.

### Study limitations

Although animal-based welfare assessments were conducted as part of the broader study, this article focuses on owner-reported management practices and therefore does not present direct equid-level welfare assessment data, which may limit interpretation of welfare outcomes. In addition, while information was collected on working hours, distance travelled, and the provision of rest breaks, detailed indicators of workload intensity, such as load weight, terrain, speed, and the duration or quality of rest periods, were not measured. Reliance on self-reported data may also be subject to recall and social desirability bias. The use of snowball sampling may have introduced selection bias and limits the generalisability of the findings beyond the study communities.

### Animal welfare implications

This study investigated the management and welfare of working equids in Limpopo Province, South Africa, where equids play an important role in transport and agricultural activities. The findings revealed significant welfare concerns, including inadequate feeding and disease management practices, limited access to veterinary services, and widespread reliance on owner-led treatment, often in the absence of accurate diagnosis or professional guidance. In addition, observations indicated frequent contraventions of existing animal welfare legislation.

Based on these findings, key next steps to improve the welfare of WEs should include targeted, context-appropriate owner education programmes focusing on nutrition, disease prevention, and early recognition of health problems. Improving the availability and accessibility of veterinary services, including mobile or community-based veterinary outreach, could help address current gaps in care. Strengthening collaboration between government agencies, animal welfare organisations, veterinary professionals, and local communities is also essential to support knowledge exchange, improve compliance with welfare legislation, and develop sustainable, locally acceptable interventions. Together, these approaches may contribute to improved welfare outcomes for working equids while supporting the livelihoods of equid-owning communities.

## Conclusion

Despite the significant contribution of WEs to livelihoods, agriculture, and transport, these animals remain at risk of compromised welfare due to a failure to meet welfare needs and inadequate access to essential services. Veterinary services are often inaccessible, or availability poorly communicated, further exacerbating welfare challenges. Findings from this study indicate that the welfare of working equids is also undermined by widespread non-compliance with existing legislation, including the Animal Protection Act (1962) and the Animal Diseases Act (1984). Such non-compliance often appears to be unintentional and linked to limited awareness of legal requirements, as well as cultural perceptions of equids as ‘beasts of burden’.

While enforcement of animal welfare legislation is important, reliance on punitive approaches alone may be ineffective and risks placing additional pressure on already marginalised communities. Improving working equid welfare is therefore likely to require a combination of education, awareness-raising, and supportive interventions alongside appropriate enforcement. Collaborative efforts involving veterinary professionals, government agencies, animal welfare organisations, social scientists, and equid owners have the potential to improve understanding of welfare and legal obligations, encourage compliance, and promote humane treatment. Such integrated approaches are more likely to deliver sustainable welfare improvements while also supporting the livelihoods of equid-owning communities.

## Supporting information

10.1017/awf.2026.10097.sm001 Molapo et al. supplementary material Molapo et al. supplementary material

## Supplementary material

The supplementary material for this article can be found at http://doi.org/10.1017/awf.2026.10097.

## Long descriptions

### Figure 1. Long description

The left panel shows a map of South Africa divided into districts. In the Northeast, the Waterberg District is highlighted in blue, while adjacent districts in the Limpopo Province are shaded in dark gray.

The right panel is a detailed map of the Waterberg District Municipality. A blue arrow from the left map points to the Mogalakwena local municipality in the Northeast of the district. The district is divided into five main local municipalities:

* Lephalale in the Northwest.

* Thabazimbi in the Southwest.

* Modimolle-Mookgophong in the central and Eastern region.

* Bela-Bela in the South.

* Mogalakwena in the Northeast.

Specific towns and survey locations are marked with black dots within these regions. In Mogalakwena, the town of Lekalakala is marked. In Modimolle-Mookgophong, the towns of Vaalwater, Mookgophong, Nylstroom, and Roedtan are identified. In Thabazimbi, the town of Thabazimbi is marked. In Bela-Bela, the town of Bela-Bela is marked. The bottom left corner of the second panel contains the text w w w dot municipalities dot co dot z a.

Navigate back to Figure 1..

### Table 1. Long description

The table consists of four columns: Variable, Level, Frequency (n), and Percentages (%).

* How often do you take your equids out for grazing?

- Once: 41 (36.94%)

- Twice: 9 (8.11%)

- Three times: 5 (4.50%)

- No grazing schedule: 56 (50.45%)

* Do you provide supplementary feeding to your equines?

- No: 103 (92.79%)

- Yes: 8 (7.21%)

* What do your equids feed mainly on?

- Crop residues: 19 (17.12%)

- Grazing: 106 (95.50%)

- Concentrates: 3 (2.70%)

- Other: 2 (1.80%)

* From where your equids get drinking water?

- Kraal: 97 (87.39%)

- Stream: 18 (16.22%)

* Where do you keep your equids at night?

- Kraal: 50 (45.05%)

- Bush: 62 (55.86%)

- Around the yard: 7 (6.31%)

Navigate back to Table 1..

### Table 2. Long description

The table is organized into four columns: Variable, Action, Frequency (n), and Percentages (%).

* Response to sick equids: 79.28% (n=88) treat it themselves, 7.21% (n=8) call local welfare or a vet, 6.31% (n=7) do nothing, 5.41% (n=6) report they don’t get sick, and 1.80% (n=2) call another owner.

* Sources for drugs: 67.57% (n=75) use agricultural co-ops, 28.83% (n=32) do not buy medicine, 4.50% (n=5) get them from neighbors, and 3.60% (n=4) use the local S P C A.

* Types of drugs commonly bought: 49.55% (n=55) buy antiparasitic drugs, 36.94% (n=41) buy antiseptics, 11.71% (n=13) are not sure, and 0.90% (n=1) buy antibiotics.

* Internal parasite treatment: 88.29% (n=98) do not treat, while 11.71% (n=13) do.

* External parasite treatment: 53.15% (n=59) do not treat, while 46.85% (n=52) do.

* Vaccination: 100% (n=111) do not vaccinate their equids.

* Procedures in the last three months: 0.90% (n=1) performed castration, while 0% performed hoof trimming or dentistry.

Navigate back to Table 2..

### Table 3. Long description

The table contains four columns: Variable, Level, Frequency n, and Percentage %.

* How often do you use your equines: Availability of work 68 (61.26%), Daily 33 (29.73%), Weekly 6 (5.41%), Seasonal 3 (2.70%), Not Applicable 1 (0.90%).

* Seasonal usage (Yes responses): Winter 79 (71.17%), Summer 68 (61.26%), Spring 29 (26.13%), Autumn 25 (22.52%).

* Average daily working hours: 3 to 6 h 86 (77.48%), Less than 2 h 21 (18.92%), 7 to 11 h 3 (2.70%), More than 11 h 0 (0%), Depends on number of loads 1 (0.90%).

* Daily travel distance: 0 to 4 km 64 (57.66%), 5 to 10 km 44 (39.64%), More than 10 km 3 (2.70%).

* Rest breaks: Yes 110 (99.10%), No 0 (0%), Not Applicable 1 (0.90%).

* Communication methods (Yes responses): Voice command 105 (94.59%), Whipping 12 (10.81%), Pull in the reins 12 (10.81%).

Navigate back to Table 3..

### Table 4. Long description

The table consists of four columns: Variable, Option, Frequency (n), and Percentages %.

The variable question is: What happens to your equids if it can’t work anymore?

The data for the options is as follows:

* Euthanasia: Frequency 1, 0.90 percent.

* Sell it: Frequency 4, 3.60 percent.

* Continue taking care until it dies: Frequency 88, 79.28 percent.

* Slaughter for meat: Frequency 0, 0 percent.

* Take it to Local S P C A: Frequency 7, 6.31 percent.

* Neglect: Frequency 5, 4.50 percent.

The total sample size is n = 111.

Navigate back to Table 4..

### Table 5. Long description

The table consists of four columns: Variable, Level, Frequency n, and Percentages %.

- For the variable ‘Do you use harness for work?’:

- Level ‘No’ has a frequency of 1 and a percentage of 0.90.

- Level ‘Yes’ has a frequency of 110 and a percentage of 99.10.

- For the variable ‘Is it appropriate for your type of work?’:

- Level ‘No’ has a frequency of 53 and a percentage of 47.75.

- Level ‘Yes’ has a frequency of 57 and a percentage of 51.35.

Navigate back to Table 5..
