# Supplementary material for: Management practices and welfare of working equids in Mogalakwena local municipality, Limpopo Province, South Africa
Source: Anim Welf. 2026 Jul 23;35:e44. doi: 10.1017/awf.2026.10097 (PMC13419466; doi:10.1017/awf.2026.10097)
Supplement: Molapo et al. supplementary material [file S0962728626100979sup001.pdf]

Management practices and welfare of working equids in Mogalakwena local municipality, Limpopo Province, South Africa: Supplementary Material

Dimakatso B Molapo, Cheryl ME McCrindle, Tulisiwe Mbombo-Dweba, James W Oguttu

https://orcid.org/0000-0001-6810-4437

University of South Africa, Department of Agriculture and Animal Health, Florida Campus, Johannesburg, 1709, South Africa

Author for correspondence: James W Oguttu, email: jwoguttu@unisa.ac.za

SI. Questionnaire guide (abbreviated version)

Household survey on management and welfare of working equids in Mogalakwena Local Municipality, Limpopo Province, South Africa
Target respondents: Households owning working equids

Section A — Location and equid type
Respondents were asked to indicate:
1. Study location (one of 14 peri-urban areas).
2. Type of working equid owned (donkey or mule).

Section B — Working patterns of equids
Respondents provided information on:
- Frequency of use (daily, weekly, seasonal, or work-dependent).
- Main seasons of use.
- Average hours worked per day.
- Approximate distance travelled per day.
- Whether rest breaks were provided.
- Primary communication methods used with equids during work (voice, reins, or whipping).

Section C — Harnessing
Respondents were asked whether:
- A harness was used during work.
- The harness was considered appropriate for the tasks performed.

Section D — Feeding, water and housing
Respondents reported on:
- Number of daily feeding events.
- Main feed types (grazing, crop residues, concentrates, or other).
- Provision of supplementary feed.
- Main drinking water source.
- Night-time housing or confinement of equids.

Section E — Disease and parasite management
Questions addressed:
- Actions taken when an equid was sick.
- Whether internal deworming was practiced.
- Who administered deworming treatments.
- Whether external parasite control was practiced.

Section F — Fate of equids at end of working life
Respondents indicated what typically happens to an equid when it can no longer work (e.g., euthanasia, sale, continued care, slaughter, transfer to SPCA, or neglect).

Section G — Access to services
Respondents were asked:
- Whether equine welfare organisations were present in their area and what services were received in the previous three months.
- Whether any government services related to equines were received.

SII. Distribution of households in the study area that owned WEs.

Location	Frequency (n)	Percentage (%)
Sekgakgapeng	19	17.12
Maroteng	17	15.32
Masehlaneng	13	11.71
Tshamahansi	12	10.81
Mosestjane	11	9.91
Moshate	8	7.21
Mozombane	6	5.41
Masodi	6	5.41
Phola Park	6	5.41
Malepetleke	5	4.50
Madiba	4	3.60
Magongoa	4	3.60
Mitchel	0	0
Mountain View	0	0
**Total**	111	100%

SIII. Distribution of working equids based on type.

Variable		Frequency(n)	Percentage (%)
Type	Donkey	404	99.26
	Mule	3	0.74
	Horse	0	0
Total		407	100

SIV. Stakeholders' interventions in equids related activities

Variable	Level	Frequency (n)	Percentage (%)
Are you receiving any services from the government that are related to equines?	No	111	100
	Yes	0	0
Are there organisations / institutions that are concerned with equine welfare in your area?	No	93	83.78
	Yes	18	16.22
If yes, what services did they provide in the last 3 months?	No services received	11	9.91
	Euthanasia	1	0.90
	Pound services	1	0.90
	Medical services	4	3.60
	Harness repair / supply	0	0
	Not applicable	94	84.68
